# Large-scale simulation of coverage and error rate tradeoffs for cancer detection in cell-free DNA whole-genome sequencing

**DOI:** 10.1093/bioinformatics/btag460

**Published:** 2026-08-21

**Authors:** Li-Ting Chen, Jeroen de Ridder, Myrthe Jager

**Affiliations:** Center for Molecular Medicine, University Medical Center Utrecht, Utrecht University, Utrecht 3584 CX, The Netherlands; Princess Máxima Center for Pediatric Oncology, Utrecht 3584 CS, The Netherlands; Oncode Institute, Utrecht 3521 AL, The Netherlands; Center for Molecular Medicine, University Medical Center Utrecht, Utrecht University, Utrecht 3584 CX, The Netherlands; Princess Máxima Center for Pediatric Oncology, Utrecht 3584 CS, The Netherlands; Oncode Institute, Utrecht 3521 AL, The Netherlands; Center for Molecular Medicine, University Medical Center Utrecht, Utrecht University, Utrecht 3584 CX, The Netherlands; Princess Máxima Center for Pediatric Oncology, Utrecht 3584 CS, The Netherlands; Oncode Institute, Utrecht 3521 AL, The Netherlands

## Abstract

**Motivation:**

Cell-free DNA (cfDNA) whole-genome sequencing (WGS) is a promising approach for detecting cancer recurrence. It enables cancer detection by identifying all tumor-derived cfDNA (ctDNA) molecules carrying somatic single nucleotide variants (sSNVs). While ideally, a sequencing platform should be highly accurate for reliable ctDNA detection, in reality, all sequencing platforms introduce sequencing errors that generate false positives indistinguishable from true SNVs. Understanding how sequencing parameters influence ctDNA detection sensitivity at low tumor fractions (TFs) in cfDNA samples is essential for guiding sequencing strategies in clinical contexts. To model cfDNA sequencing for tumor detection, which contains asymmetric noise and multiple interacting parameters, analytical modeling is intractable, motivating large-scale parallelized simulation.

**Results:**

We developed a simulation framework to generate *in silico* cfDNA data across 10 cancer types. In total, 480 million cfDNA samples were simulated from tumor WGS profiles. Overall, the lowest detectable TF differs substantially between cancer types under identical sequencing conditions due to variations in mutational load. For cancers with high mutational load, 3× coverage with low-error techniques reliably detects TFs below 0.1%. In contrast, cancers with low mutational load require at least six-fold higher coverage to achieve comparable detection thresholds. Increasing sequencing quality scores from Q30 to Q55 at 30× coverage further enhances sensitivity, enabling detection of TFs as low as 1 × 10^−5^. This study provides a comprehensive framework for optimizing sequencing parameters, offering valuable guidance for tailoring future technology development for specific cancer types and clinical applications.

**Availability and implementation:**

The code is publicly available at https://github.com/UMCUGenetics/cfdetect/tree/main.

## 1 Introduction

Early identification of cancer recurrence and minimal residual disease (MRD) substantially improves therapeutic efficacy and overall survival across nearly all cancer types (Crosby *et al.* 2022). Liquid biopsy-based approaches, such as the analysis of tumor-derived cell-free DNA (ctDNA) in blood, offer a minimally invasive means to monitor cancer dynamics. ctDNA fragments recapitulate the genomic profile (i.e. mutations) of the tumor cells and thus serve as highly specific biomarkers (Cisneros-Villanueva *et al.* 2022, Pascual *et al.* 2022). Nevertheless, ctDNA detection remains technically challenging due to the typically low ctDNA fractions (tumor fraction; TF) in MRD and early recurrence (Azad *et al.* 2020, Bredno *et al.* 2022). Digital-droplet PCR (ddPCR) assays targeting individual mutations are routinely used in the clinic, but their sensitivity is limited by the depth of available cell-free DNA (cfDNA), which is only ∼8000 genome equivalents (GEs) per 10 ml blood sample (Alborelli *et al.* 2019). Whole-genome sequencing (WGS), in contrast, leverages the breadth of the genome by interrogating all somatic single-nucleotide variations (sSNVs) simultaneously, thereby expanding the number of mutation sites for surveillance up to six orders of magnitude. In this tumor-informed genome-wide approach, all SNVs in the tumor can act as biomarkers to distinguish between tumor-derived ctDNA and healthy cfDNA, substantially increasing theoretical detection sensitivity. However, WGS platforms have inherent error rates and false positive errors are often indistinguishable from true SNVs. Several methods have been developed to counteract this error rate, such as duplex sequencing, addition of unique molecular identifiers (UMIs), and rolling circle amplification (RCA)-based consensus sequencing (Chen *et al.* 2025). These methods improve base-calling accuracy but typically do so at the expense of coverage, thereby increasing the chance of missing true mutations. Understanding the tradeoff between sequencing coverage and error rate is crucial for developing cost-effective liquid biopsy tests tailored to specific tumor types.

## 2 Approach overview

**Figure 1:**
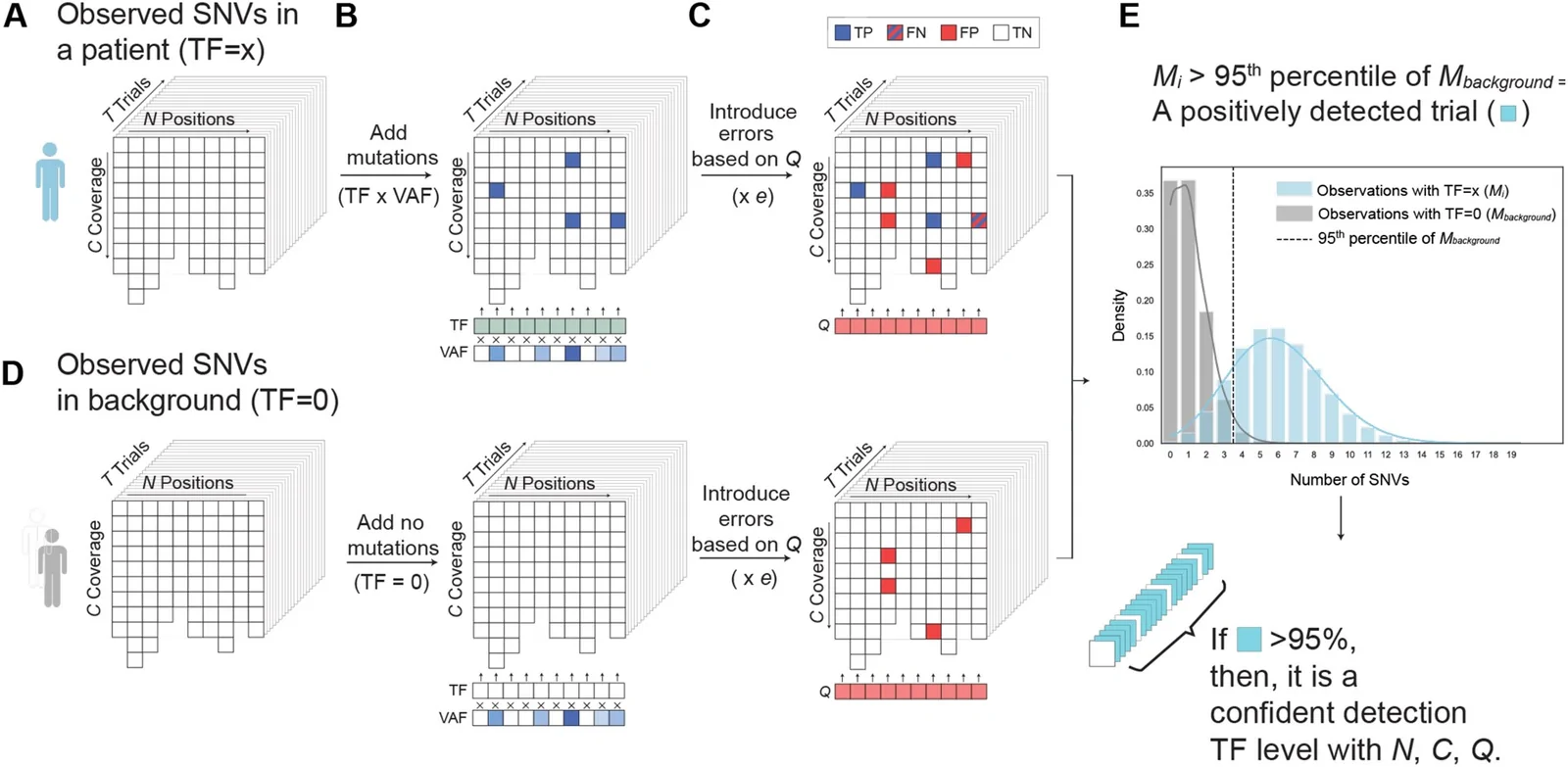
Simulating ctDNA mutation detection for various tumor fractions under a range of sequencing conditions. (A) In each patient, the number of SNVs is extracted as *N*. Based on coverage *C*, the number of reads overlapping sSNV positions (*N)* is calculated and randomly distributed for *T* trials (*T* = 100). (B) Mutations are added to each *N* by multiplying VAF of the sSNV and TF. (C) Erroneous SNV observations are added based on Q score, resulting in FP and FN observations. The total observed mutation count is denoted as *Mi*. (D) A background level with the same Q score but with TF = 0 is simulated to derive *M_background_*. (E) Based on the 95th percentile of *M_background_*, we determine a confidence level of 95% (black dashed line), above which a trial is identified as positive. If >95 of the 100 trials are positive, the TF is determined to be confident detected in this patient. The lowest TF level confidently detected is defined as the patient-level lowest detectable tumor fraction (p-ldTF).

In this manuscript, we conducted a comprehensive simulation study to investigate the effect of sequencing quality score (*Q*) and sequencing coverage (*C*) on the ability to detect low TFs with tumor-informed genome-wide SNV detection in cfDNA across 10 tumor types. Sequencing quality score (*Q*) is the Phred score, defined as −10 × log10(*E*), where *E* is the sequencing error rate, and sequencing coverage (*C*) is defined as the average number of reads mapping to any location in the genome. *In silico* cfDNA WGS data were simulated to mimic real-world sequencing data. To this end, WGS data from 10 tumor types in the PCAWG dataset were analyzed to quantify the number of sSNV and their variant allele frequencies (VAF) in each genome (Fig. S1A, C, D, available as supplementary data at *Bioinformatics* online) (ICGC/TCGA Pan-Cancer Analysis of Whole Genomes Consortium 2020). From this data, 450–500 patient sSNV profiles per tumor type were interpolated (Fig. S1B, available as supplementary data at *Bioinformatics* online). Based on the sSNV count (*N*) of each patient profile and a given coverage (in the range 0.05×–30×), the number of reads overlapping sSNV positions across the genome was calculated (Fig. 1A). For each read overlapping a sSNV position, a mutated allele or a wild-type allele was assigned using a binomial draw with a probability equal to the VAF multiplied by the simulated TF (Fig. 1B). Sequencing *Q* (in the range Q20–Q55) was then used to introduce false positive and false negative observations, contributing to the final number of observed sSNV (*Mi*) (Fig. 1C). For each condition, which represents a unique combination of *N*, *C*, and *Q*, *T *= 100 independent simulated cfDNA tests were performed. Background sSNV observations in individuals without cancer (TF = 0) were also simulated (Fig. 1D), creating a distribution *M_background_* for each condition (Fig. 1E). A positive cfDNA test was defined when the simulated *M_ic_* exceeds the 95th percentile of *M_background_*. We defined the patient-level lowest detectable tumor fraction (p-ldTF) as the lowest TF level where >95% of the simulated cfDNA tests were positive. Repeating this process for each patient profile allowed identification of detectable TFs under various hypothetical sequencing conditions.

## 3 Materials and methods

### 3.1 Calling somatic point mutations and determining variant allele frequencies in PCAWG cohort of selected tumor types

Matched tumor-normal WGS data from patients with cancer from the PCAWG cohort are gathered and processed as described in (Priestley *et al.* 2019, Roepman *et al.* 2021, Martínez-Jiménez *et al.* 2023). A subset of samples was selected from 10 representative tumor types; 50 samples were selected when there were between 50 and 100 samples available in PCAWG, and 100 samples when there were ≥100 samples. No statistical methods were used to predetermine sample size. Somatic variants were processed with the Hartwig analytical pipeline SAGE (v2.2) as described in (Priestly *et al.* 2019, Martínez-Jiménez *et al.* 2023), using Strelka (v1.0.146; Saunders *et al.* 2012), BWA (v0.7.17; Li and Durbin 2009) and GATK Haplotype Caller (v3.8.0; McKenna *et al.* 2010). The adjusted variant allele frequency (VAF) based on tumor purity inference was reported by Hartwig analytical pipeline PURPLE (v2.14) as described in (Priestly *et al.* 2019, Mart**í**nez-Jim**é**nez *et al.* 2023), so that the variant allele frequencies of each sSNV represent the VAF within the tumors only regardless of tumor purity of the isolated samples. Details and settings of these tools can be found at https://github.com/hartwigmedical/hmftools. sSNVs only (excluding INDELs) in autosomal chromosomes (chromosome 1–22) that have PASS quality filters were retained for the purpose of this study.

### 3.2 Simulating patient sSNV profiles with interpolation

The distribution of sSNV counts across patients is very wide and uneven (Fig. S1A, available as supplementary data at *Bioinformatics* online). To obtain a more continuous distribution, simulated patient profiles were simulated based on the true patient sSNV profiles available in PCAWG. For each tumor type, sSNV counts were interpolated along the sorted patient sSNV counts using cubic spline interpolation (Fig. S6, available as supplementary data at *Bioinformatics* online). Interpolated values that exceeded the range of the original data were excluded. The interpolated sSNV count distribution was compared to the original patient group sSNV count distribution with a Kolmogorov–Smirnov (KS) test to confirm no statistical differences were inadvertently introduced (Table S3, available as supplementary data at *Bioinformatics* online). To assign each simulated sSNV with a VAF, VAFs were randomly sampled with replacement from the sample with the closest sSNV count. This yields, for each simulated patient profile k a set of Nk SNVs with associated VAFs a*s*


(1) $$\{v_{kj}\},\;j=1,\ldots ,N_{k}$$

### 3.3 Simulation framework for tumor-informed cfDNA SNV detection

We conducted a simulation study to quantify how sequencing coverage and sequencing accuracy jointly determine the detectability of a tumor using tumor-informed, genome-wide SNV detection in cfDNA sequencing data. Sequencing accuracy was parameterized by the Phred quality score


(2) $$Q=-10\cdot \log_{10}(E)$$

where *E* denotes the average error rate. Sequencing coverage was represented as *C*, corresponding to the average genome-wide coverage. *E* and *C* were evaluated across a discrete grid of values. *E* was varied between 1.00** × **10^**−**^^2^ and 3.16** × **10^**−**^^6^, *E* ∈ {0.01, 0.00316, 0.001, 0.00066, 0.000316, 0.0001, 0.0000316, 0.00001, 0.00000316}, corresponding to *Q* ranging from Q20 to Q55. *C* was evaluated at *C* ∈ {0.05×, 0.25×, 0.5×, 1.0×, 1.5×, 3.0×, 5.0×, 10.0×, 15.0×, 30.0×} genome equivalents. Results for 10.0×, 15× and 30.0× coverage of skin melanoma and colorectal carcinoma patients were omitted due to exceeding the maximum allocated computing time of 7 days.

For a given patient profile with *N_k_* sSNVs and sequencing depth *C*, the number of sSNV loci covered per simulated cfDNA test, $K_{k}$, was generated according to the sequencing depth. When *C* < 1, was sampled from a binomial distribution, assuming that each sSNV locus had an independent probability *C* of being covered. When C≥1, per-locus coverage was sampled from a normal distribution centered on C with standard deviation C/2, summed across all $N_{k}$ sSNV loci, and rounded to the nearest integer:


(3) $$K_{k}=\{\begin{array}{ll} K_{k}∼\mathrm{Binomial}(N_{k},C), & C<1,\\ K_{k}=\mathrm{round}(\sum_{j=1}^{N_{k}}X_{j,k}),X_{j,k}∼N(C,{\left(\frac{C}{2}\right)}^{2}), & C\geq 1. \end{array}\;$$

For a simulated tumor fraction TF, each covered sSNV *j* was assigned a tumor-derived mutation allele according to a Bernoulli process with success probability


(4) $$p_{ij}=\text{TF}\cdot v_{ij}$$

For simulated cfDNA test *i* and sSNV *j*, *X_ij_* indicates whether a mutant or a wild-type allele is observed at that sSNV locus, with *X_ij_* = 1 indicating a mutant allele and *X_ij_* = 0 indicating a wild-type allele. The total number of true mutant allele observations in simulated test *i*, denoted *Si*, was obtained by summing over all covered sSNVs:


(5) $$S_{i}=\sum_{j=1}^{K_{k}}X_{ij},\;X_{ij}∼\text{Bernoulli}(p_{kj})$$

Sequencing errors were subsequently introduced to account for false-negative and false-positive observations, with miscalling true mutant alleles with probability *E* and miscalling wild-type alleles as mutant with probability *E*/3. When a mutant allele is missequenced as another base, it will be counted as non-mutant, with a probability of *E*. While the wildtype allele is missequenced exactly as the base of the mutant allele, which is one of the three missequenced options, to be counted as a mutation, with error rate *E*/3. In the simulation experiment, we assume that all base alterations have an equal error probability, without considering platform-specific or context-dependent error signatures. The total number of observed mutant alleles *M_i_* in cfDNA test *i* was therefore given by


(6) $$M_{i}=S_{i}-{\text{FN}}_{i}+{\text{FP}}_{i}$$

where FN_*i*_ and FP_*i*_ denote false-negative and false-positive observations arising from sequencing errors.

We evaluated 100 logarithmically spaced tumor fraction (TF) values ranging from 0.001% to 100% for every combination of simulated patient sSNV profile, sequencing depth (*C*), and sequencing error rate (*E*). For each parameter combination, 100 independent simulated cfDNA tests *T* were performed.

Background distributions *M_background_* were generated with 100 cfDNA tests for cancer-free condition (TF = 0) in the same manner for each sequencing parameter combination. A simulated cfDNA test was classified as a *positive test* if the observed mutant count exceeded **τ** which we set to the 95th percentile of the background distribution. For each patient profile and sequencing parameter combination, we defined as


(7) $$p-\text{ldTF}=\min\{\text{TF}:\;\mathrm{Pr}(M_{i}>\tau )>0.95\}$$

Here, Pr(*Mi* >** τ**) represents the empirical fraction of simulated cfDNA tests whose observed mutant count exceeds the background-derived detection threshold. In other words, patient-level lowest detectable tumor fraction (p-ldTF) corresponds to the minimum TF level at which >95% of simulated cfDNA tests yield an observed mutant count exceeding the background-derived detection threshold (τ).

### 3.4 Tumor-level lowest detectable tumor fraction determination

Tumor-level lowest detectable tumor fraction (t-ldTF) was defined as a summary measure of tumor detectability with cfDNA tests across patients within a given tumor type. While p-ldTF captures the minimum tumor fraction required for reliable detection in an individual patient, t-ldTF reflects population-level detectability within a tumor type.

Specifically, t-ldTF corresponds to the tumor fraction at which at least 95% of patients with a specific tumor type are detectable by the cfDNA test. Operationally, t-ldTF was defined as the 95th percentile of the distribution of p-ldTF for that tumor type, such that at this tumor fraction, no >5% of patients remain below the detection threshold. This definition can be expressed mathematically as


(8) $$t-\text{ldTF}={\text{quantile}}_{0.95}(\{{p-\text{ldTF}}_{k}\},\;k=1,\ldots ,N_{\text{patients}})$$

### 3.5 Simulating cfDNA tumor fraction based on cancer volume using surface growth model

To demonstrate the relationship between cfDNA tumor fraction in cfDNA and underlying tumor volume, we utilized a simplistic surface growth model (Murphy *et al.* 2016), simulating residual tumor growth following surgical removal. The surface growth model assumes tumor growth is directly proportional to nutrient and oxygen access, which correlates linearly with the tumor’s surface area as a sphere structure. Tumor radius is calculated from tumor volume as *r* = (3 V/4π)^1/3^, and tumor surface area *S* *=* 4πr^2^. At each time step, the growth volume is *kS*. We initialized simulations with a starting volume of ∼1000 cells (∼1 100 000 µm³). By assuming an initial doubling time as 2 days, the volume expansion constant (k) was set to 2.4. Recognizing cell death as concurrent with tumor proliferation, we assumed a cell death rate of 50%, which gives rise to tumor-derived cfDNA. We calculated the cfDNA tumor fraction as the ratio of dead tumor cells to the total number of dead blood cells, under the assumption that all cells produce the same amount of cfDNA upon death. Daily blood cell death was set at 0.5 billion cells (Sender and Milo 2021), and incorporated a physiological variance of 5% with a normal distribution to reflect natural fluctuations in systemic cellular turnover.

The simulation extended over 300 days across two simulated cancer patient scenarios. cfDNA tumor fractions derived from this model were subsequently compared to the lowest detectable tumor fractions achievable by various sequencing techniques with approximated quality score listed in parentheses, including Nanopore (Q20), Illumina NovaSeq (Q30), PacBio (Q40), Ultima (Q40), or a hypothetical future technique (Q50) and coverage depths (ranging from 0.05× to 30×).

### 3.6 Statistics and visualization

All statistical analyses were performed using Python (v3.10.12), numpy (v1.26.0), and scipy (v1.11.3). For comparison of the distribution between original and interpolated patient sSNV distributions, Kolmogorov–Smirnov tests were used to test significance.

All plots were generated using pandas (v1.5.3), matplotlib (v3.8.0), and seaborn (v0.13.2) in Python (v3.10.12). Logarithmic scaling was applied where appropriate. Figure preparation and layout were performed using Adobe Illustrator.

## 4 Results

### 4.1 Error rate and coverage affect lowest detectable tumor fraction in cancer cfDNA samples

**Figure 2:**
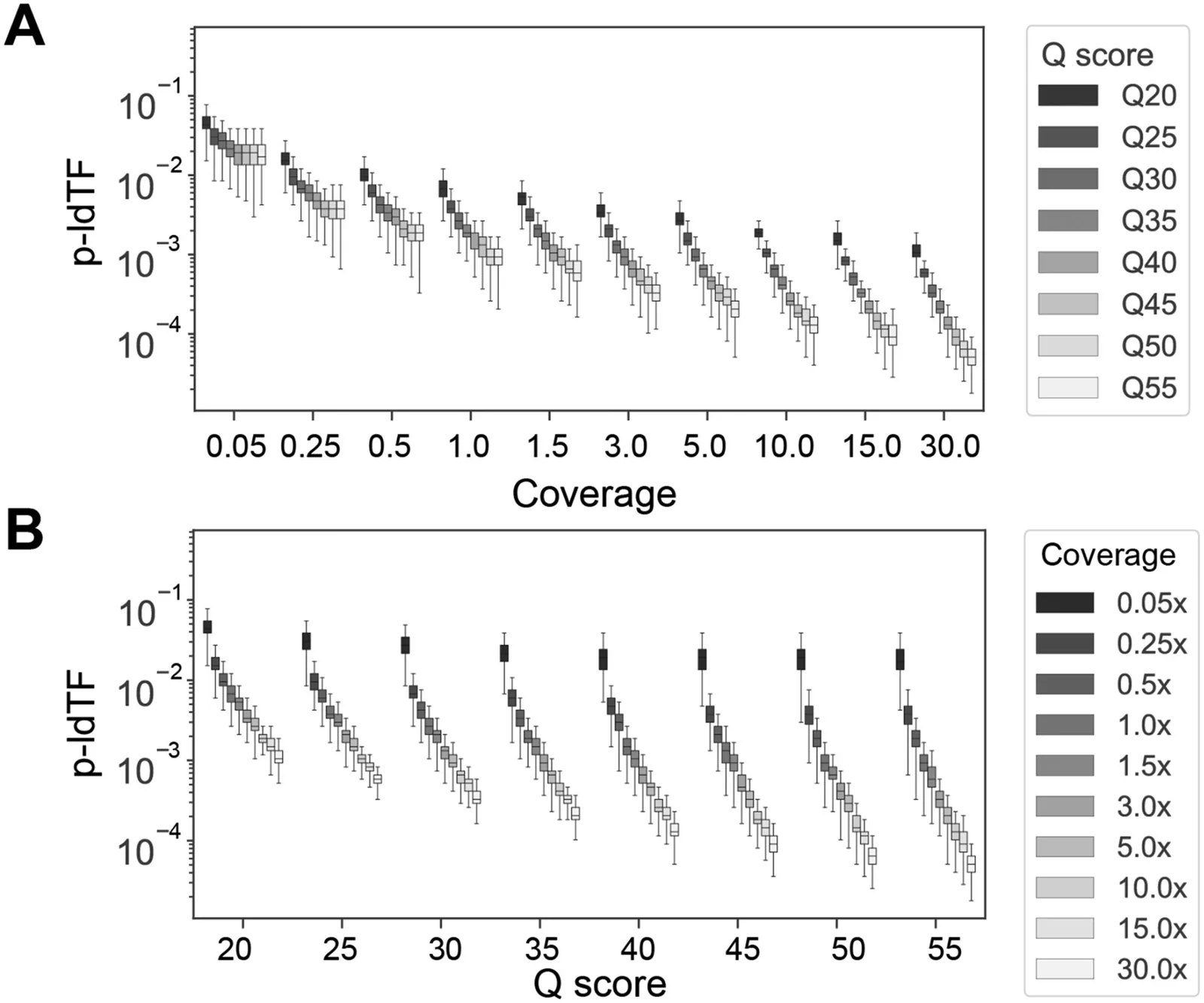
Effects of increasing Q score and coverage on lowest detectable tumor fraction in esophageal cancer patients. (A) The effect of sequencing error rates from Q20 to Q55 on lowest detectable tumor fraction with various sequencing coverage (0.05×, 0.25×, 0.5×, 1.0×, 1.5×, 3.0×, 5.0×, 10.0×, 15.0×, and 30.0×) for esophageal cancer patients represented as grouped boxplots (whiskers extended to the first and third quartile). (B) The effect of sequencing coverages (0.05×, 0.25×, 0.5×, 1.0×, 1.5×, 3.0×, 5.0×, 10.0×, 15.0×, and 30.0×) on lowest detectable tumor fraction with various sequencing error rates, Q20 to Q55, for esophageal cancer patients represented as grouped boxplots (whiskers extended to the first and third quartile).

Increasing the Q score generally improved the p-ldTF (Fig. 2A, Fig. S2, available as supplementary data at *Bioinformatics* online), by reducing *M_background_* and thus lowering the noise. However, this effect was not linear. Improving from a low Q to a medium Q overall had more effect on the p-ldTF than improving from a medium to a high Q (Fig. S2, available as supplementary data at *Bioinformatics* online). For example, improving from Q20 to Q35 at 3× coverage in esophageal cancer reduced the mean p-ldTF ∼4.4-fold, while reducing the error rate from Q40 to Q55 at 3× coverage only decreased the mean p-ldTF 2.2-fold (Fig. 2A). This demonstrates that approaches that improve the Q score (such as RCA- and UMI-based consensus sequencing) are especially beneficial for sequencing techniques with a low Q score. Notably, increasing the Q score to Q50 and beyond, at coverages below 1.0×, did not effectively improve p-ldTF, due to the fact that true observations of sSNVs were often missed (Fig. 2A). This emphasizes that error reduction alone is insufficient unless adequate sequencing depth is achieved as well.

Increasing sequencing depth improved the p-ldTF in general as well, by enhancing the differences between *M_i_* and *M_background_* (Fig. 2B, Fig. S3, available as supplementary data at *Bioinformatics* online). As opposed to improving the Q score, the effect of improving coverage was evident across all Q score levels. However, the impact of increasing coverage on p-ldTF was more pronounced at low coverages than at high coverages (Fig. 2B, Fig. S3, available as supplementary data at *Bioinformatics* online). For example, 0.05× is too low for adequate TF detection, as the p-ldTF is still above 0.001, even at 30× coverage. Unlike Q scores, which are intrinsic to a sequencing platform, coverage is determined by sequencing throughput and can be adapted as needed, though in practice it is typically constrained by cost per sample. Overall, we conclude that increasing Q score is only effective when the coverage is sufficient (Fig. 2A), while increasing coverage is effective for detection regardless of the Q score (Fig. 2B).

### 4.2 The lowest detectable tumor fraction is highly influenced by tumor mutational burden

**Figure 3:**
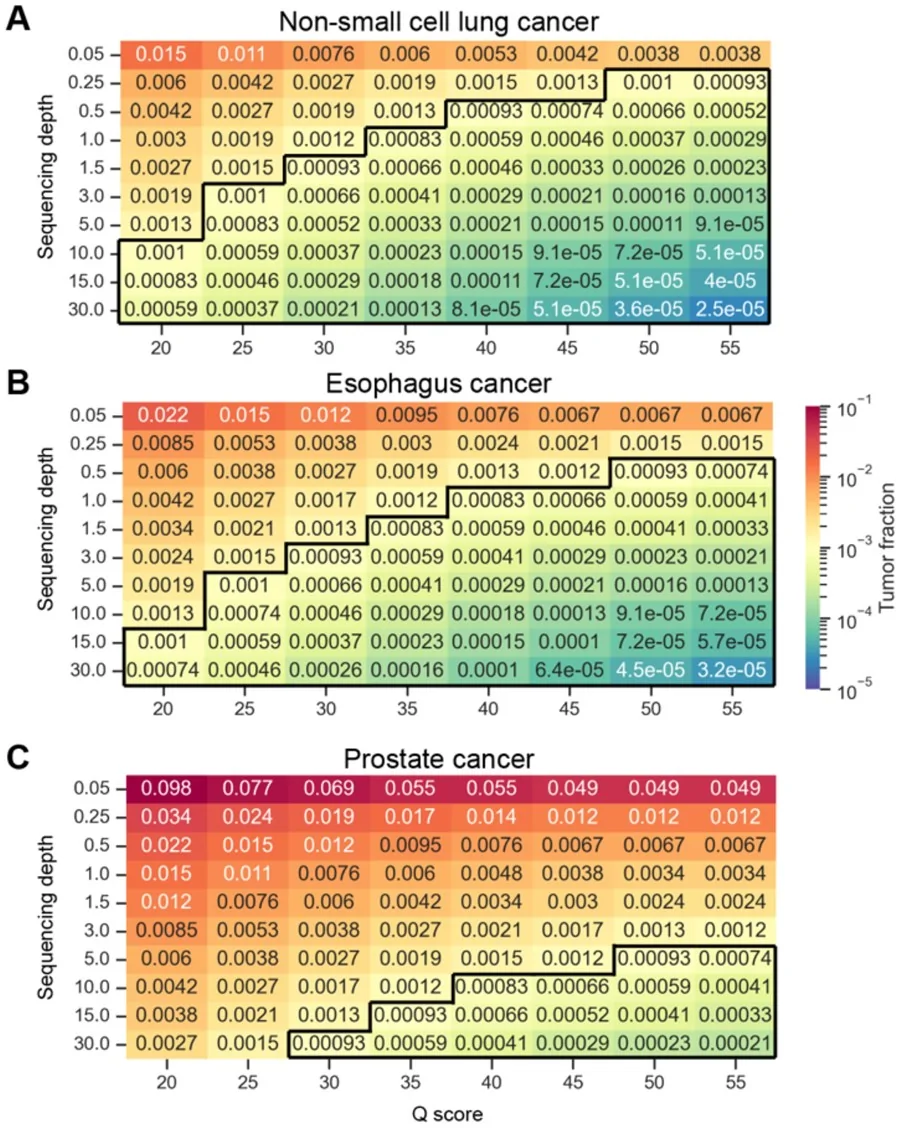
Lowest detectable tumor fractions of varying sequencing conditions across 10 cancer types. The heatmaps depict the minimum tumor fraction detectable (rainbow gradient colormaps in log scale) as a function of both sequencing depth (0.05× to 30×, y-axis) and Q scores (Q20 to Q55, x-axis) for (A) non-small-cell lung cancer, (B) esophageal cancer, (C) prostate cancer. Conditions that detect ldTF < 0.001 are outlined by a black line.

The effects of changes in coverage and Q score on the p-ldTF also largely depend on the tumor mutational burden (TMB), the total number of somatic mutations in a tumor’s genome (Figs S2 and S3, available as supplementary data at *Bioinformatics* online). The number of sSNVs was highly variable between and within tumor types, ranging from 1994 in a prostate cancer patient to 862 986 in a skin melanoma patient in our cohort (Table 1, Table S1, Fig. S1, available as supplementary data at *Bioinformatics* online). We summarized the lowest detectable tumor fraction in a tumor type (t-ldTF) as the lowest TF at which >95% of the patients were detected. Examining the t-ldTFs across various tumor types at the highest sequencing accuracy (Q55), non-small cell lung cancer and esophageal cancer (ranked third and fourth in TMB) demonstrate exceptionally low t-ldTFs of 3.1** × **10^**−**^^5^ and 2.2** × **10^**−**^^5^, respectively, at 30× coverage (Fig. 3, Table S2, available as supplementary data at *Bioinformatics* online). Skin melanoma and colorectal carcinoma (ranked first and second in TMB) are even detectable with TFs as low as 8.7 × 10^−5^ and 1.1 × 10^**−**^^4^, respectively, at 5× coverage (Fig. S4, available as supplementary data at *Bioinformatics* online). In contrast, tumor types with lower TMBs, such as prostate cancer, require higher coverage and sequencing quality for adequate detection. For example, the t-ldTF in prostate cancer at 5× coverage with Q55 is 6.9 × 10^**−**^^4^, which is eight times higher than for skin melanoma (Fig. 3, Fig. S4, available as supplementary data at *Bioinformatics* online). This illustrates how TMB, and by extension tumor type, can affect the ability to detect a tumor from cfDNA sequencing at a specific tumor fraction even in similar sequencing scenarios.

**Table 1:** Summary statistics of simulated patient sSNV profiles across tumor types.

Tumor type	Unique simulated patient sSNV profiles (*n*)	sSNVs per patient (median)	sSNVs per patient (min)	sSNVs per patient (max)
Skin melanoma	493	68 698	2549	862 986
Non-small-cell lung cancer	495	32 564	1225	124 716
Esophageal cancer	491	19 988	5857	79 263
Colorectum carcinoma	476	16 034	6520	2 284 354
Stomach cancer	495	12 854	220	398 135
Hepatocellular carcinoma	484	11 983	3612	58 371
Ovarian cancer	483	6985	479	40 301
Breast cancer	470	5330	1251	29 693
Pancreas carcinoma	457	5100	2187	52 457
Prostate cancer	451	2677	309	230 403

For each tumor type, the number of unique simulated profiles (*n*) and the distribution of sSNVs (median, min, max), based on interpolated sSNV distributions, is reported.

### 4.3 Cancer recurrence detection based on existing sequencing technique landscape

**Figure 4:**
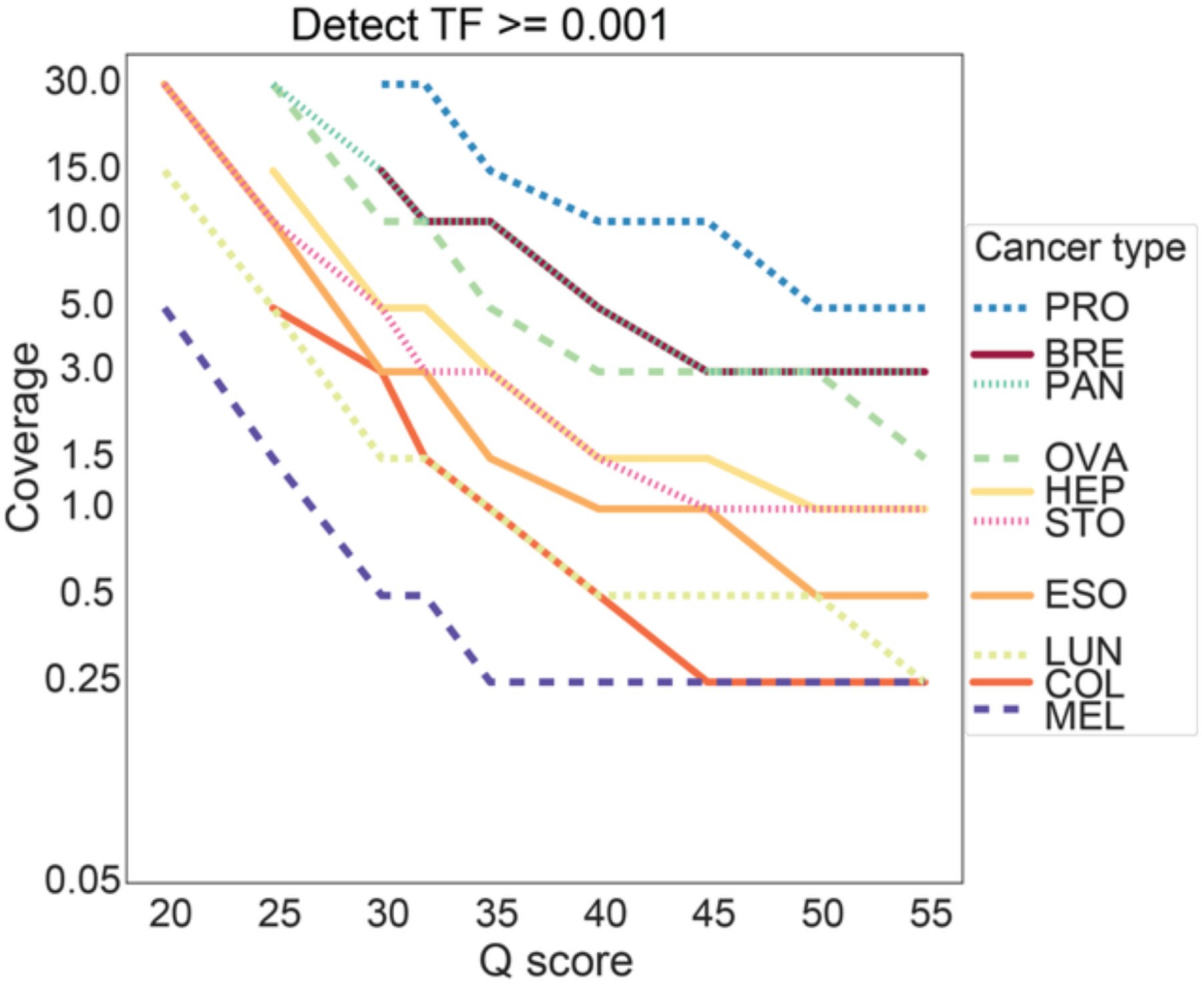
Minimum sequencing requirements for achieving ldTF of 0.001 across cancer types. Each line represents, per Q score, the minimal coverage required to detect a tumor fraction of 0.001 for a given cancer type. Coverage is plotted on a log scale. Abbreviations: BRE, breast cancer; COL, colorectum carcinoma; ESO, esophagus cancer; HEP, hepatocellular carcinoma; LUN, non-small-cell lung cancer; MEL, skin melanoma; OVA, ovarian cancer; PAN, pancreas carcinoma; PRO, prostate cancer; STO, stomach cancer.

Building on these theoretical findings, we evaluated current sequencing technologies, including Illumina  NovaSeq X (NovaSeq^TM^ X ), PacBio Onso (Onso system specification sheet, 2025), Ultima UG100 (Ultima 2024;   Ultima Genomics, Inc, 2025), and Oxford Nanopore Technologies (ONT) (Nanopore sequencing accuracy), with simulations. These sequencing technologies offer distinct combinations of coverage, sequencing error rates, and cost (Table 2). Some methods provide extremely high throughput and low cost per base. To efficiently leverage these technologies batching of samples using multiplexed libraries is required, making it difficult to apply in point-of-care settings. Other methods enable fast turn-around time without the need for multiplexing, but have overall lower throughput and higher cost per base such as ONT sequencing. In current cfDNA-based diagnostic tests, tumor fractions around 0.005 are generally achievable; however, detection below this level remains inconsistent across methods and represents an important area for further improvement (Deveson *et al.* 2021). We assessed the possible combinations of sequencing parameters to achieve one level lower than what is generally achievable, at 0.001 (Fig. 4, Fig. S5, available as supplementary data at *Bioinformatics* online). For high-mutation cancers, such as esophageal cancer, sequencing with Q20 at 30× can detect TFs ≥0.001, making it feasible to implement a test based on the ONT sequencing platform, thus potentially benefiting from its fast turnaround time and enabling novel clinical applications (Fig. 4). For the same ldTF level, Illumina NovaSeq achieves similar sensitivity at ≥3× coverage, while Ultima UG100 and PacBio Onso require only ≥1.0× coverage. For low-mutation cancers like ovarian cancer, on the other hand, achieving TF ≤ 0.001 requires at least Q25 at 30×. With ONT’s current accuracy, even two full PromethION flowcells, equivalent to 30×, is insufficient to detect TF ≤ 0.001 (Table 2). Hence, the optimal sequencing strategy largely depends on tumor type and the required ldTF (Fig. 3).

**Table 2:** Error rate and cost of commercially available sequencing techniques.^a^

Sequencing technique	Q score	List price per unit (euro)^b^	WGS coverage per unit	Cost per 1× coverage (euro)^b^
ONT PromethION	20	955	15×	66
Illumina NovaSeq X	30	9888	720×	14
PacBio Onso	40	995	38×	26
Ultima Genomics UG100	40^c^	2500	780×	3.3
Roche SBX duplex	40	Not disclosed	310× (per hour)	Not disclosed

a ONT, Oxford Nanopore Technologies. References: (NovaSeq^TM^ X and NovaSeq X Plus Sequencing Systems), (Onso system specification sheet 2025), (Ultima Genomics, Inc. 2025), *(*Sequencing by expansion (SBX)/AGBT 2025 virtual IR event), (Nanopore sequencing accuracy).

b Prices reflect consumables only, accessed on 1 November 2025.

c Claimed Q40 > 85% on spec sheet D1000987 Rev. 01 released in September 2024 (Ultima 2024); while claimed Q 30>85% on spec sheet D1001006 Rev.02, released in February 2025 (Ultima Genomics, Inc. 2025).

### 4.4 Patient-specific sequencing throughput can be determined based on patient specific tumor profile to enable optimal cancer detection

**Figure 5:**
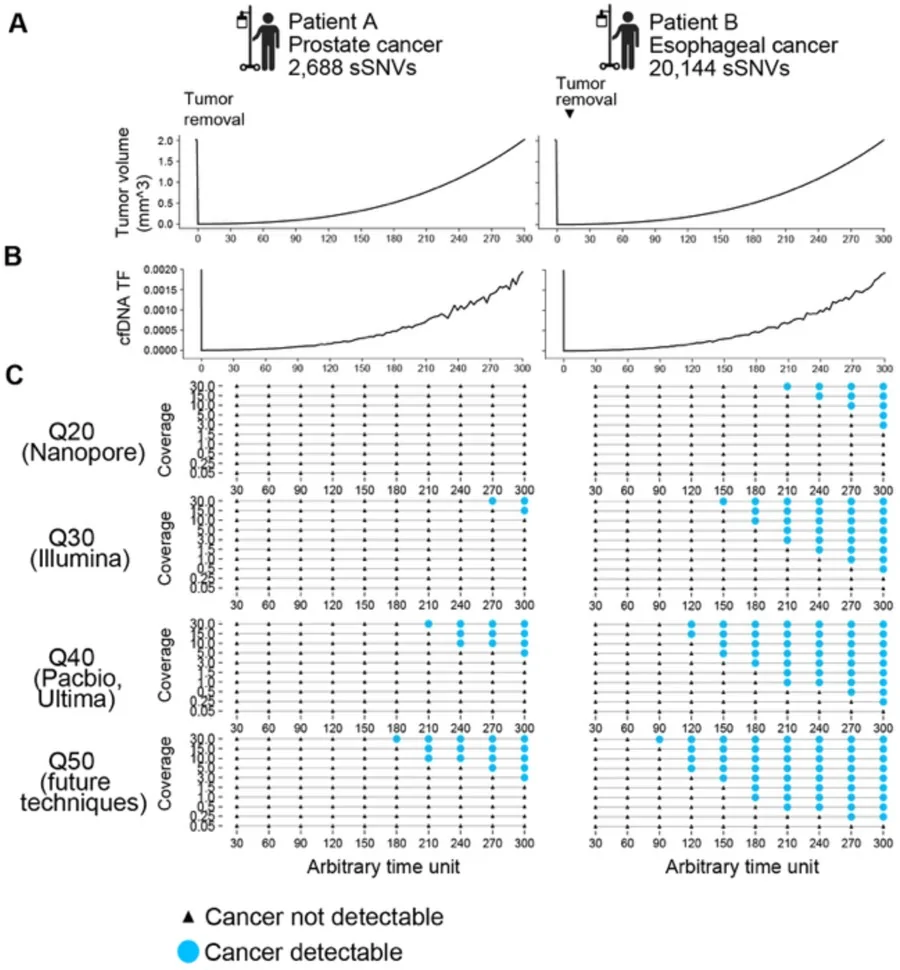
Tumor evolution, cfDNA tumor fraction dynamics and detectability with various coverages of representing different sequencing techniques across two patients. (A) Overview of the two patients: Patient A (prostate cancer) and Patient B (esophageal cancer) and the hypothetical tumor volume (y-axis) representing residual tumor growth over time (x-axis) after surgical removal. (B) cfDNA TF over time in the two patients correlates to tumor volume while with different noise representing physiological influence on cfDNA measurements. (C) Tumor detectability in cfDNA over arbitrary time unit in these patients with different sequencing technologies: Q20 (Nanopore), Q30 (Illumina), Q40 (PacBio and Ultima), and Q50 (future techniques), and various coverage between 0.05× and 30×. Blue circles indicate detectable cancer, while black triangles indicate undetectable cancer.

So far, we have shown examples of how combinations of sequencing depth and error rate influence tumor detectability in a hypothetical patient cohort at a single timepoint. However, tumors evolve and TFs change accordingly over time (Fig. 5). To illustrate the importance of tumor evolution, we simulated tumor growth across a 300 artificial time unit (a.t.u.) timespan following surgical removal of the tumor in two patients, one with prostate cancer (2688 sSNVs) and one with esophageal cancer (20 144 sSNVs). We then determined the impact of *C*, *Q*, and *N* on the earliest detectable timepoint of cancer recurrence.

Using ONT sequencing, recurrence remained undetectable in the prostate cancer patient even with 30× coverage at 300 a.t.u. In the patient with esophageal cancer, however, ONT allowed cancer recurrence detection at 30× after 210 a.t.u. For both patients, achieving cancer detection 60 a.t.u. earlier generally required increasing the Q score by 10. Reassuringly, further improvements in sequencing quality, such as achieving Q50 or higher, could enhance detection sensitivity at earlier a.t.u.s, particularly when combined with adequate coverage.

It is important to note that there are biological variations in cfDNA abundance in different tumor types even at comparable clinical tumor stages. Therefore, the ability to detect lower TFs does not necessarily translate into earlier detection. To translate our findings into clinical practice, it is crucial to correlate detected TF levels with disease status, to ensure that TF measurements can serve as reliable diagnostic and prognostic indicators.

## 5 Discussion

Our results demonstrate that tumor detectability in cfDNA is shaped by multiple factors, including tumor type, sequencing platform, and coverage. Taking these factors into consideration, sequencing strategies can be tailored for individual patients using our simulation framework. In the short term, sequencing strategies should focus on achieving sufficient coverage to maximize detection sensitivity, while improvements in Q score represent a longer-term path to further enhance sensitivity, particularly at low tumor fractions. For ONT, increasing Q scores from Q20 to Q30 reduces the ldTF by around 2.5-fold at all coverages. For Illumina NovaSeq, PacBio Onso, and Ultima UG100, a similar improvement of 10 in Q score will halve the ldTF at the same coverage. Because our simulations assume uniform sequencing error rates, overlap between mutational and context-specific error profiles may increase effective false-positive rates, whereas distinct profiles may further improve discrimination between true sSNVs and sequencing artifacts. In the further future, incorporating tumor type-specific mutational profiles (Alexandrov *et al.* 2020) with sequence context-specific error rates (Stoler and Nekrutenko 2021, Liu-Wei *et al.* 2024) could therefore further improve distinguishing true sSNVs from sequencing errors.

In summary, tumor-informed genome-wide SNV detection in cfDNA enables the detection of minimal trace of tumor at unprecedentedly low ranges. While previous studies have overlooked error rates when deriving the theoretical minimal detectable TFs (van der Pol and Mouliere 2019, Zviran *et al.* 2020), we demonstrate that error rates play a critical role in determining the lowest detectable TFs and warrant greater attention. Our study provides a systematic framework for quantifying the combined impact of Q score, coverage, and tumor mutational burden on tumor detectability. By integrating these simulations with comparisons of current and future sequencing technologies, we provide actionable guidance for selecting and developing sequencing strategies tailored to specific clinical contexts.

## Supplementary Material

btag460_Supplementary_Data

## References

[btag460-B1] Alborelli I , Generali D, Jermann P et al Cell-free DNA analysis in healthy individuals by next-generation sequencing: a proof of concept and technical validation study. Cell Death Dis 2019;10:534. 31296838 10.1038/s41419-019-1770-3 PMC6624284

[btag460-B2] Alexandrov LB , Kim J, Haradhvala NJ et al; PCAWG Consortium. The repertoire of mutational signatures in human cancer. Nature 2020;578:94–101. 32025018 10.1038/s41586-020-1943-3 PMC7054213

[btag460-B3] Azad TD , Chaudhuri AA, Fang P et al Circulating tumor DNA analysis for detection of minimal residual disease after chemoradiotherapy for localized esophageal cancer. Gastroenterology 2020;158:494–505.e6. 31711920 10.1053/j.gastro.2019.10.039 PMC7010551

[btag460-B4] Bredno J , Venn O, Chen X et al Circulating tumor DNA allele fraction: a candidate biological signal for multicancer early detection tests to assess the clinical significance of cancers. Am J Pathol 2022;192:1368–78. 35948080 10.1016/j.ajpath.2022.07.007

[btag460-B5] Chen L-T , Jager M, Rebergen D et al Nanopore-based consensus sequencing enables accurate multimodal tumor cell-free DNA profiling. Genome Res 2025;35:886–99. 39805703 10.1101/gr.279144.124 PMC12047234

[btag460-B6] Cisneros-Villanueva M , Hidalgo-Pérez L, Rios-Romero M et al Cell-free DNA analysis in current cancer clinical trials: a review. Br J Cancer 2022;126:391–400. 35027672 10.1038/s41416-021-01696-0 PMC8810765

[btag460-B7] Crosby D , Bhatia S, Brindle KM et al Early detection of cancer. Science 2022;375:eaay9040. 35298272 10.1126/science.aay9040

[btag460-B8] Deveson IW , Gong B, Lai K et al; SEQC2 Oncopanel Sequencing Working Group. Evaluating the analytical validity of circulating tumor DNA sequencing assays for precision oncology. Nat Biotechnol 2021;39:1115–28. 33846644 10.1038/s41587-021-00857-z PMC8434938

[btag460-B9] ICGC/TCGA Pan-Cancer Analysis of Whole Genomes Consortium. Pan-cancer analysis of whole genomes. Nature 2020;578:82–93. 32025007 10.1038/s41586-020-1969-6 PMC7025898

[btag460-B10] Li H , Durbin R. Fast and accurate short read alignment with Burrows-Wheeler transform. Bioinformatics 2009;25:1754–60. 19451168 10.1093/bioinformatics/btp324 PMC2705234

[btag460-B11] Liu-Wei W , van der Toorn W, Bohn P et al Sequencing accuracy and systematic errors of nanopore direct RNA sequencing. BMC Genomics 2024;25:528. 38807060 10.1186/s12864-024-10440-w PMC11134706

[btag460-B12] Martínez-Jiménez F , Movasati A, Brunner SR et al Pan-cancer whole-genome comparison of primary and metastatic solid tumours. Nature 2023;618:333–41. 37165194 10.1038/s41586-023-06054-z PMC10247378

[btag460-B13] McKenna A , Hanna M, Banks E et al The genome analysis toolkit: a MapReduce framework for analyzing next-generation DNA sequencing data. Genome Res 2010;20:1297–303. 20644199 10.1101/gr.107524.110 PMC2928508

[btag460-B14] Murphy H , Jaafari H, Dobrovolny HM et al Differences in predictions of ODE models of tumor growth: a cautionary example. BMC Cancer 2016;16:163. 26921070 10.1186/s12885-016-2164-x PMC4768423

[btag460-B15] Oxford Nanopore Technologies plc. Oxford Nanopore sequencing accuracy. https://nanoporetech.com/platform/accuracy (27 February 2025, date last accessed).

[btag460-B16] Illumina, Inc. Technical note: Quality scores for Next-Generation Sequencing. 2011. https://emea.illumina.com/content/dam/illumina/gcs/assembled-assets/marketing-literature/novaseq-x-series-spec-sheet-m-us-00197/novaseq-x-series-specification-sheet-m-us-00197.pdf (27 February 2025, date last accessed).

[btag460-B17] Pacific Biosciences of California, Inc. Onso System Specification Sheet. 2023. https://3genes.com/wp-content/uploads/2023/09/Onso-specification-sheet.pdf (27 February 2025, date last accessed).

[btag460-B18] Pascual J , Attard G, Bidard F-C et al ESMO recommendations on the use of circulating tumour DNA assays for patients with cancer: a report from the ESMO precision medicine working group. Ann Oncol 2022;33:750–68. 35809752 10.1016/j.annonc.2022.05.520

[btag460-B19] van der Pol Y , Mouliere F. Toward the early detection of cancer by decoding the epigenetic and environmental fingerprints of cell-free DNA. Cancer Cell 2019;36:350–68. 31614115 10.1016/j.ccell.2019.09.003

[btag460-B20] Priestley P , Baber J, Lolkema MP et al Pan-cancer whole-genome analyses of metastatic solid tumours. Nature 2019;575:210–6. 31645765 10.1038/s41586-019-1689-y PMC6872491

[btag460-B21] Roepman P , de Bruijn E, van Lieshout S et al Clinical validation of whole genome sequencing for cancer diagnostics. J Mol Diagn 2021;23:816–33. 33964451 10.1016/j.jmoldx.2021.04.011

[btag460-B22] Saunders CT , Wong WSW, Swamy S et al Strelka: accurate somatic small-variant calling from sequenced tumor–normal sample pairs. Bioinformatics 2012;28:1811–7. 22581179 10.1093/bioinformatics/bts271

[btag460-B23] Sender R , Milo R. The distribution of cellular turnover in the human body. Nat Med 2021;27:45–8. 33432173 10.1038/s41591-020-01182-9

[btag460-B24] Roche. Sequencing by Expansion (SBX)/AGBT. 2025. https://assets.roche.com/f/176343/x/f3f0d645e5/20250220_sbx-sequencing-ir-event.pdf (3 January 2026, date last accessed).

[btag460-B25] Stoler N , Nekrutenko A. Sequencing error profiles of Illumina sequencing instruments. NAR Genom Bioinform 2021;3:lqab019. 33817639 10.1093/nargab/lqab019 PMC8002175

[btag460-B8044301] Ultima Genomics, Inc. Specification sheet: UG 100™ Sequencing Platform, The Power of Genomics at Scale. 2024. https://cdn.sanity.io/files/l7780ks7/production/d82b05dc0310d76cb66974d11ee93ceab8664c84.pdf (2 Januray 2026, date last accessed).

[btag460-B26] Ultima Genomics, Inc. Application note: Germline Whole-Genome Sequencing on the UG 100^TM^. 2025. https://cdn.sanity.io/files/l7780ks7/production-2024/f8fd8ef0ac9c81a625c52397dbb21f760ae4a6bc.pdf (27 March 2025, date last accessed).

[btag460-B27] Zviran A , Schulman RC, Shah M et al Genome-wide cell-free DNA mutational integration enables ultra-sensitive cancer monitoring. Nat Med 2020;26:1114–24. 32483360 10.1038/s41591-020-0915-3 PMC8108131

