# Supplementary material for: Large-scale simulation of coverage and error rate tradeoffs for cancer detection in cell-free DNA whole-genome sequencing
Source: Bioinformatics. 2026 Aug 21;42(Suppl 2):btag460. doi: 10.1093/bioinformatics/btag460 (PMC13501291; doi:10.1093/bioinformatics/btag460)
Supplement: Supplementary file 1 [file btag460_supplementary_data.pdf]

Supplemental materials of manuscript:

Large-scale simulation of coverage and error rate trade-offs for cancer detection in cell-free DNA whole-genome sequencing

Li-Ting Chen[1,2,3*], Jeroen de Ridder[1,2,3*], and Myrthe Jager[1,2,3*]

[1]Center for Molecular Medicine, University Medical Center Utrecht, Utrecht University, 3584 CX Utrecht, The Netherlands [2]Princess Máxima Center for Pediatric Oncology, 3584 CS, Utrecht, the Netherlands [3]Oncode Institute, 3521 AL, Utrecht, the Netherlands

*To whom correspondence should be addressed.

Supplemental Figures

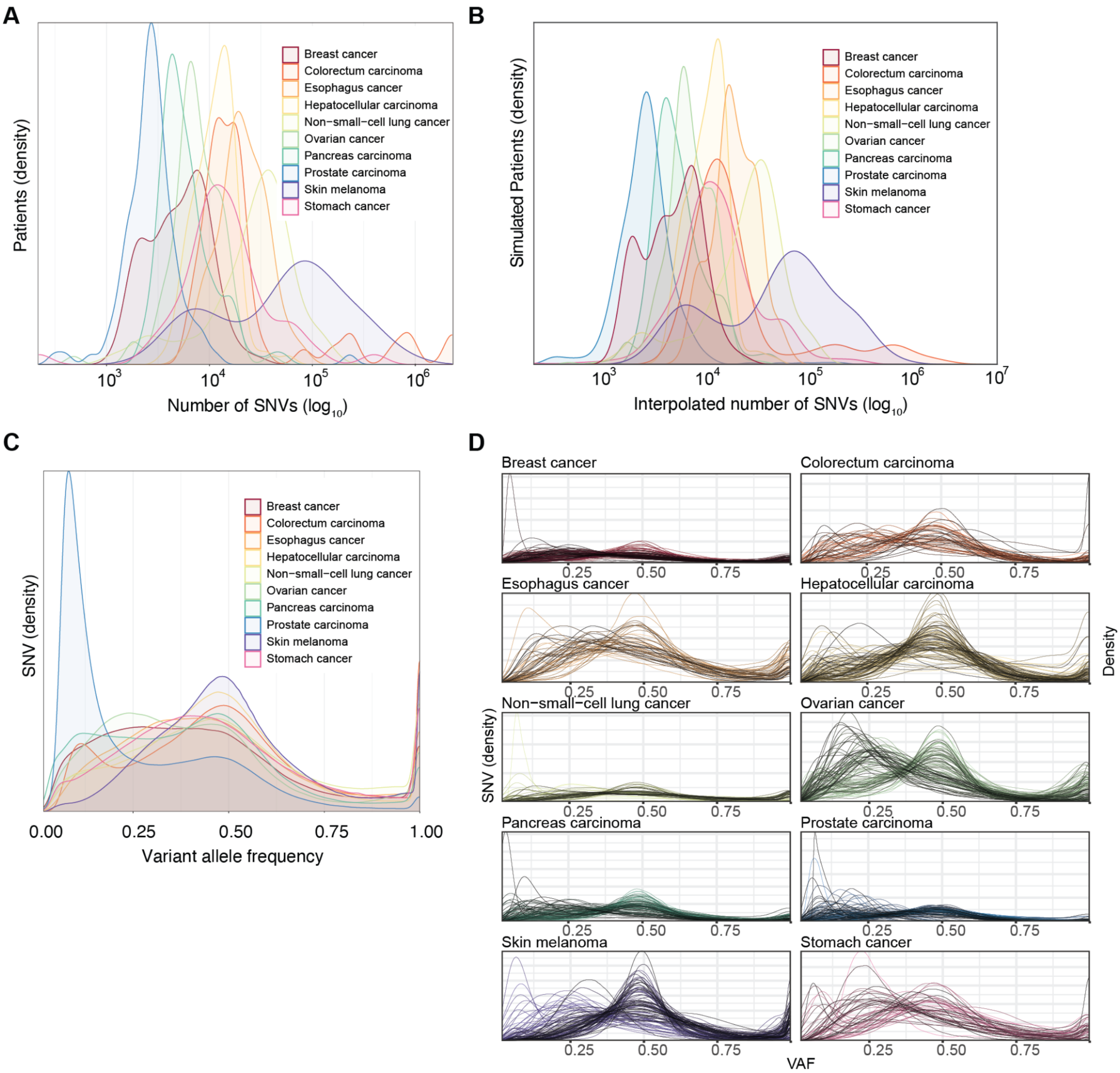

Supplementary Figure S1. SNV and VAF distribution per tumor type and per patient in PCAWG dataset and data used in the simulation study.

(A) Distribution of number of SNVs among patients in PCAWG dataset (x-axis, log scale), highlighting variability in mutation frequency between tumor types. (B) Distribution of interpolated SNV count (x-axis, log scale) per cancer types utilized in the simulations, recapitulating the distribution in PCAWG dataset in panel A. (C) Distribution of VAF across SNVs among PCAWG dataset, showing the variability between tumor types. (D) VAF distribution of SNVs for individual samples stratified by cancer type in PCAWG dataset. Each curve represents a sample, with density indicating the distribution of VAF values.

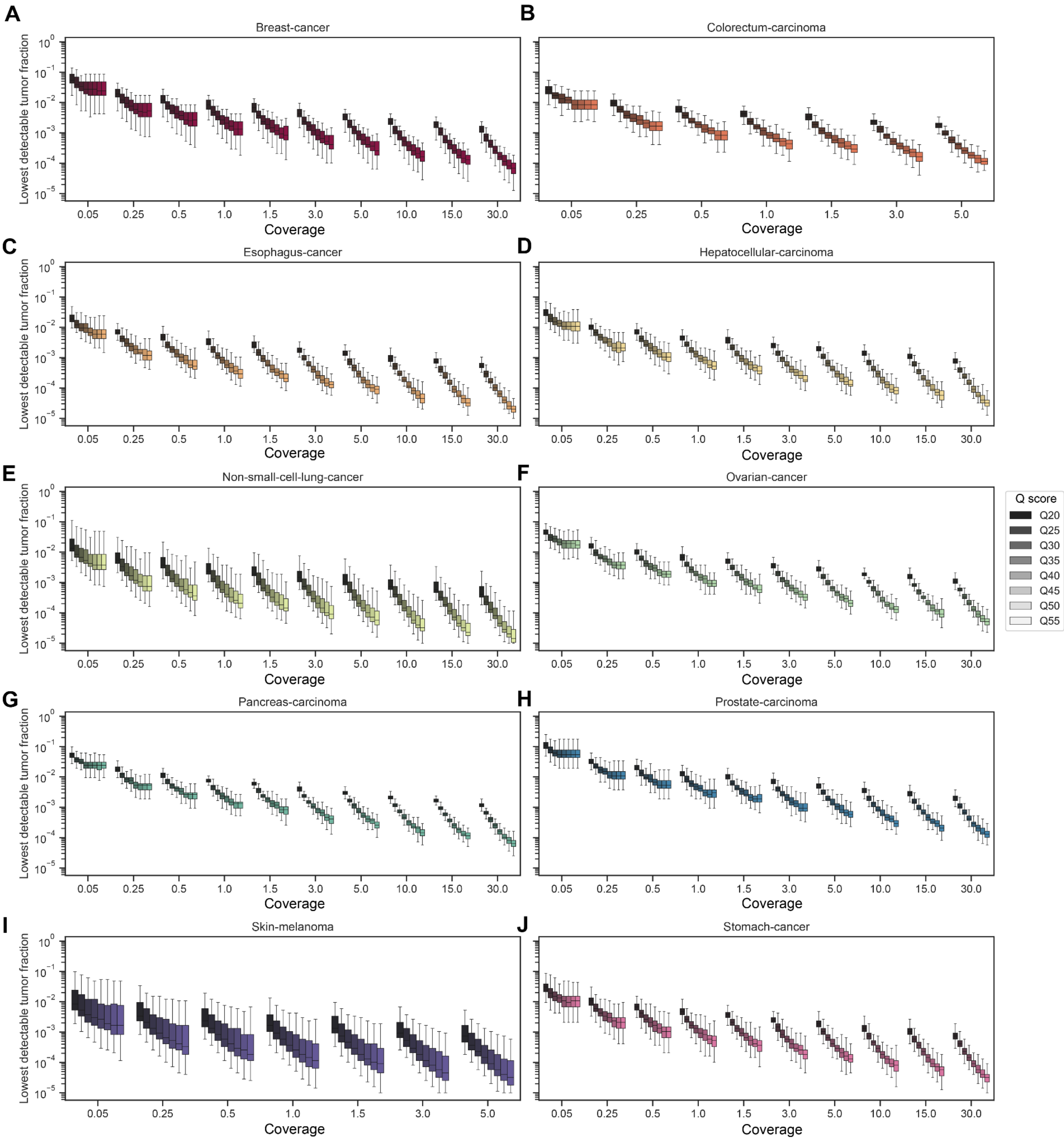

Supplementary Figure S2. Relationship between error rates and the lowest detectable tumor fraction with various sequencing depth in ten cancer types.

The plots illustrate the minimum tumor fraction detectable (y-axis, log scale) as a function of Q scores (grouped bars: Q20, Q25, Q30, Q35, Q40, Q45, Q50, and Q55) comparing between different sequencing depth (x-axis, spanning from 0.05x to 30x) for (A) breast cancer, (B) colorectal carcinoma, (C) esophageal cancer, (D) hepatocellular carcinoma, (E) non-small-cell lung cancer, (F) ovarian cancer, (G) pancreatic carcinoma, (H) prostate carcinoma, (I) skin melanoma, and (J) stomach cancer. Detectability varies across cancer types, reflecting the influence of genomic features of different tumors combined with sequencing conditions.

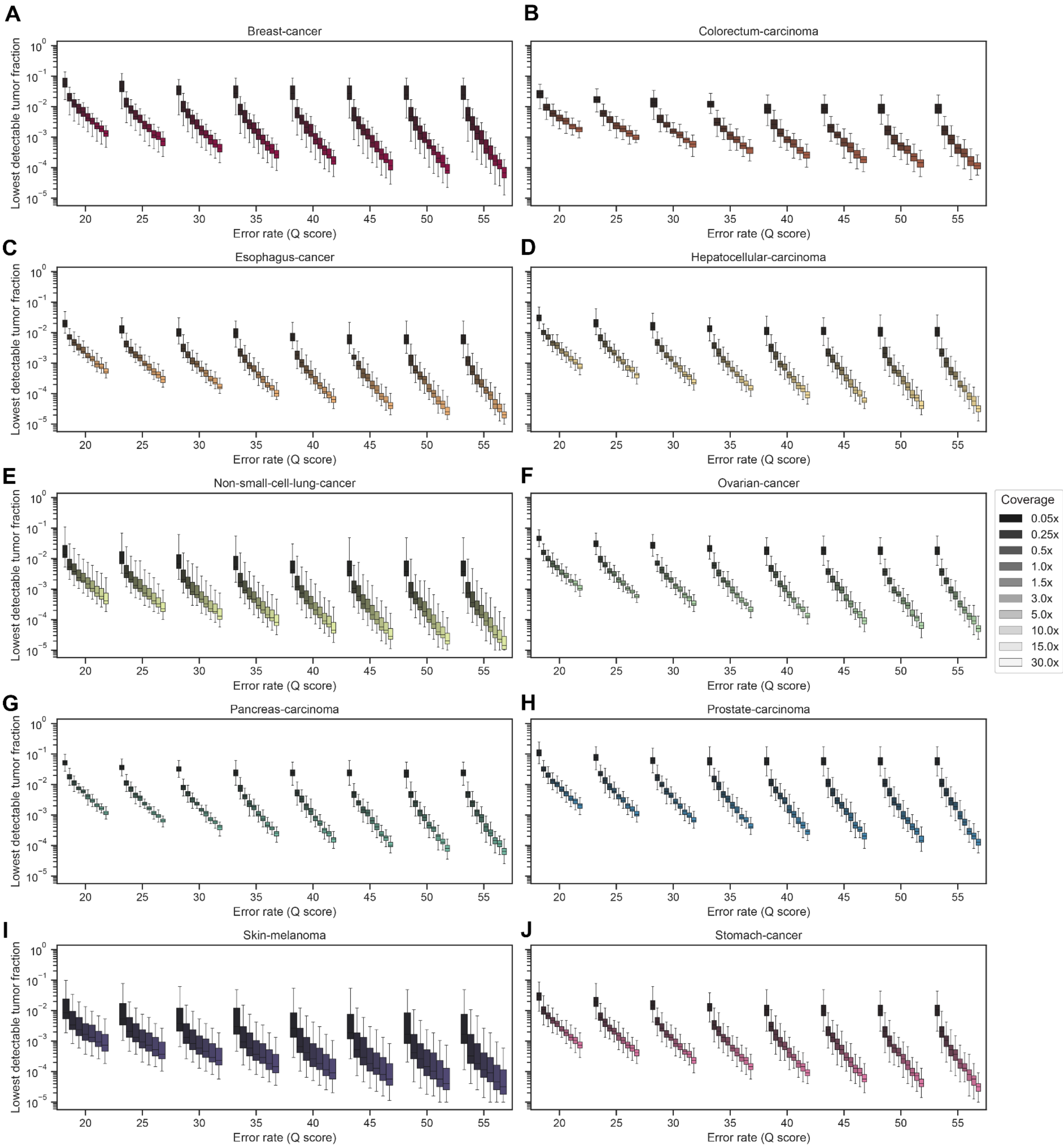

Supplementary Figure S3. Relationship between sequencing depths and the lowest detectable tumor fraction with various error rates in ten cancer types.

The plots illustrate the minimum tumor fraction detectable (y-axis, log scale) as a function of sequencing depths (grouped bars: 0.05x, 0.25x, 0.5x, 1.0x, 1.5x, 3.0x, 5.0x, 10.0x, 15.0x and 30.0x) comparing between different sequencing depth (x-axis, spanning from Q20 to Q55) for (A) breast cancer, (B) colorectal carcinoma, (C) esophageal cancer, (D) hepatocellular carcinoma, (E) non-small-cell lung cancer, (F) ovarian cancer, (G) pancreatic carcinoma, (H) prostate carcinoma, (I) skin melanoma, and (J) stomach cancer. Detectability varies across cancer types, reflecting the influence of genomic features of different tumors combined with sequencing conditions.

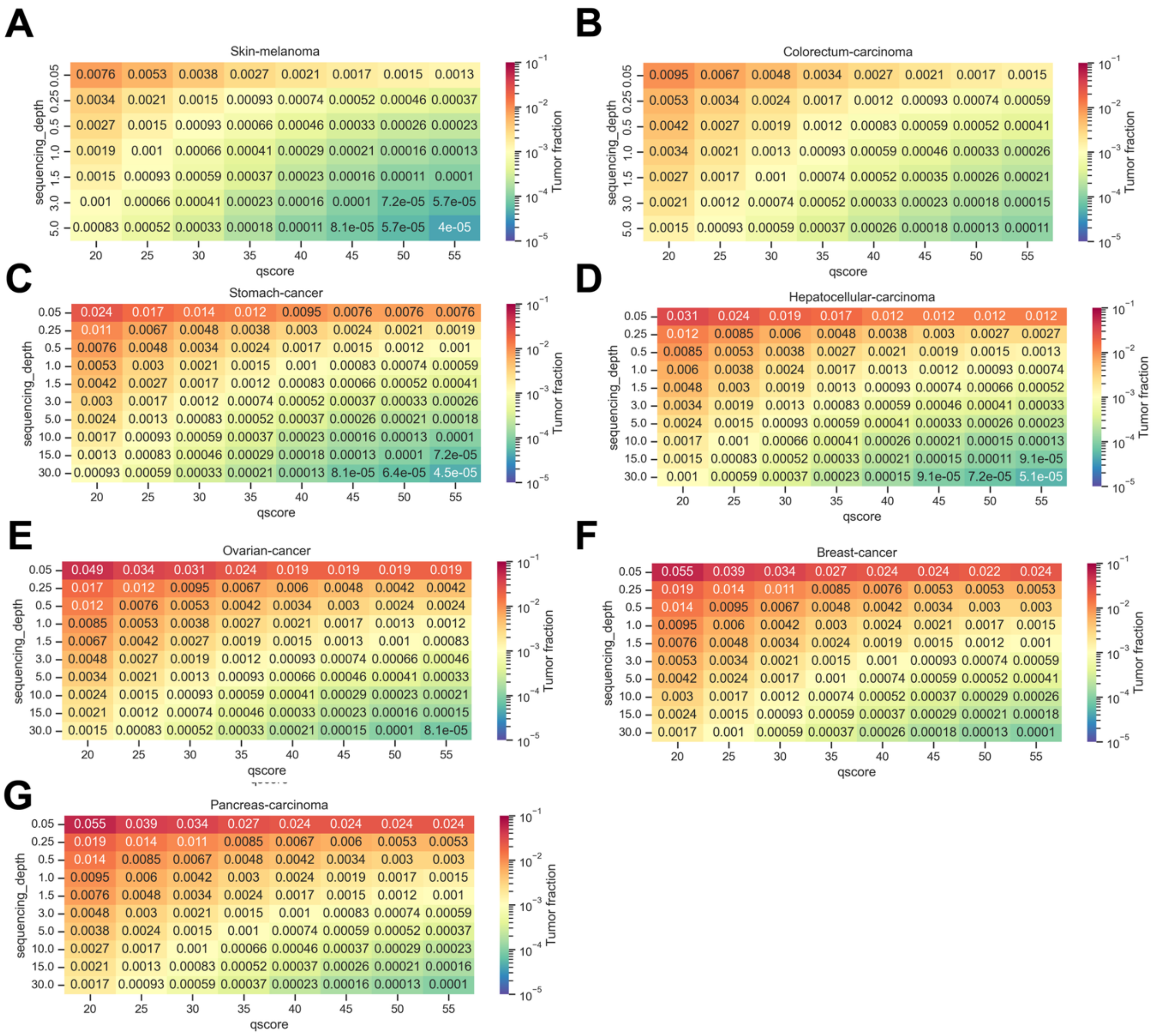

Supplementary Figure S4. Lowest detectable tumor fractions of varying sequencing conditions across cancer types.

The heatmaps depict the minimum tumor fraction detectable (rainbow gradient colormaps in log scale) as a function of both sequencing depth (0.05x to 30x, y-axis) and Q scores (Q20 to Q55, x-axis) for (A) skin melanoma, (B) colorectal carcinoma, (C) stomach cancer, (D) hepatocellular carcinoma, (E) ovarian cancer, (F) breast cancer, and (G) pancreatic carcinoma.

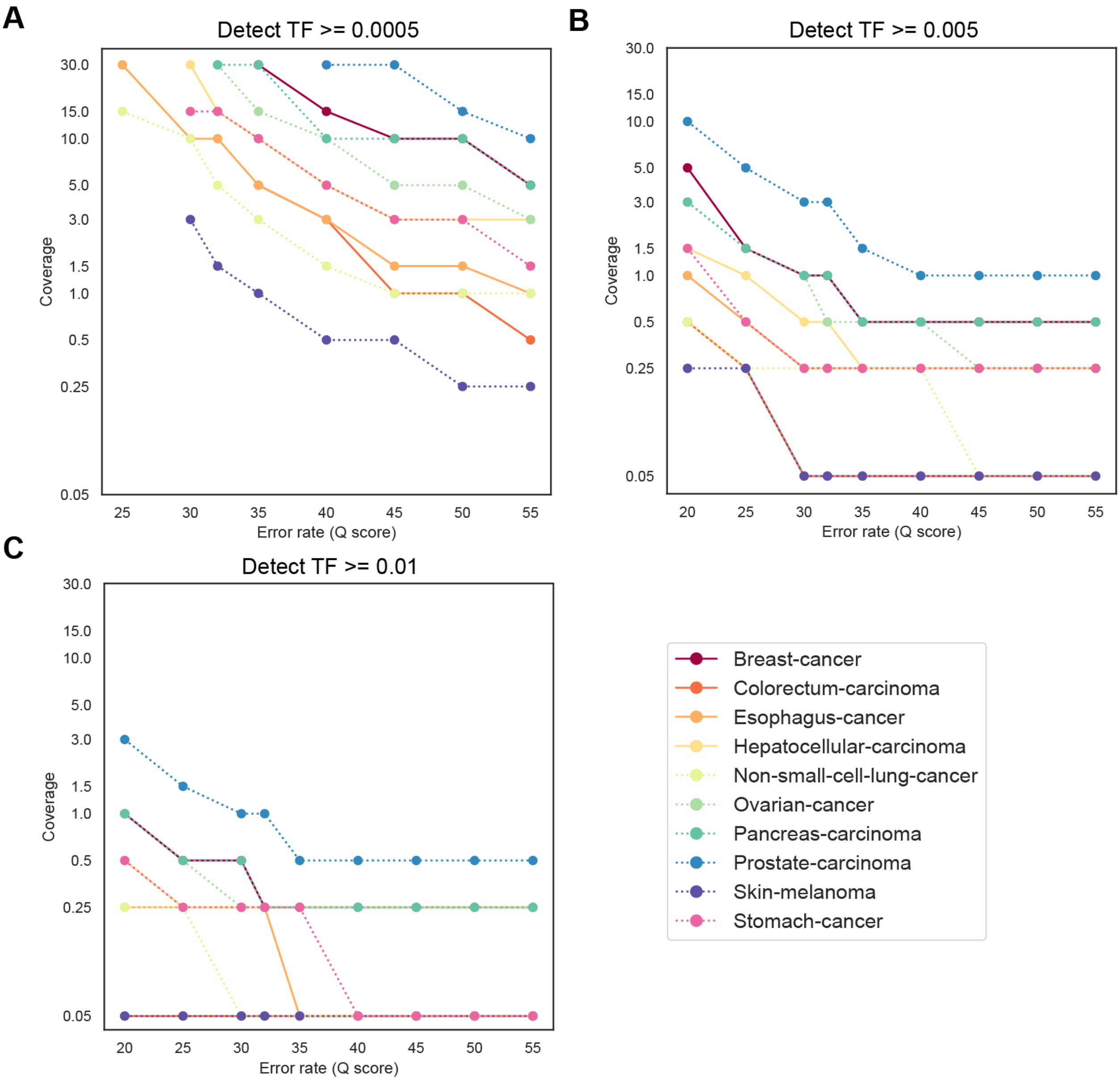

Supplementary Figure S5. Minimum sequencing requirements for achieving a TF detection limit of (A) 0.0005, (B) 0.005, (C) 0.01 across cancer types.

Each line represents per sequencing error rate (Q score), the minimal coverage required to detect a tumor fraction of 0.001 for a given cancer type. Coverage is plotted on a log scale (inverted y-axis), and higher Q scores indicate lower sequencing error rates.

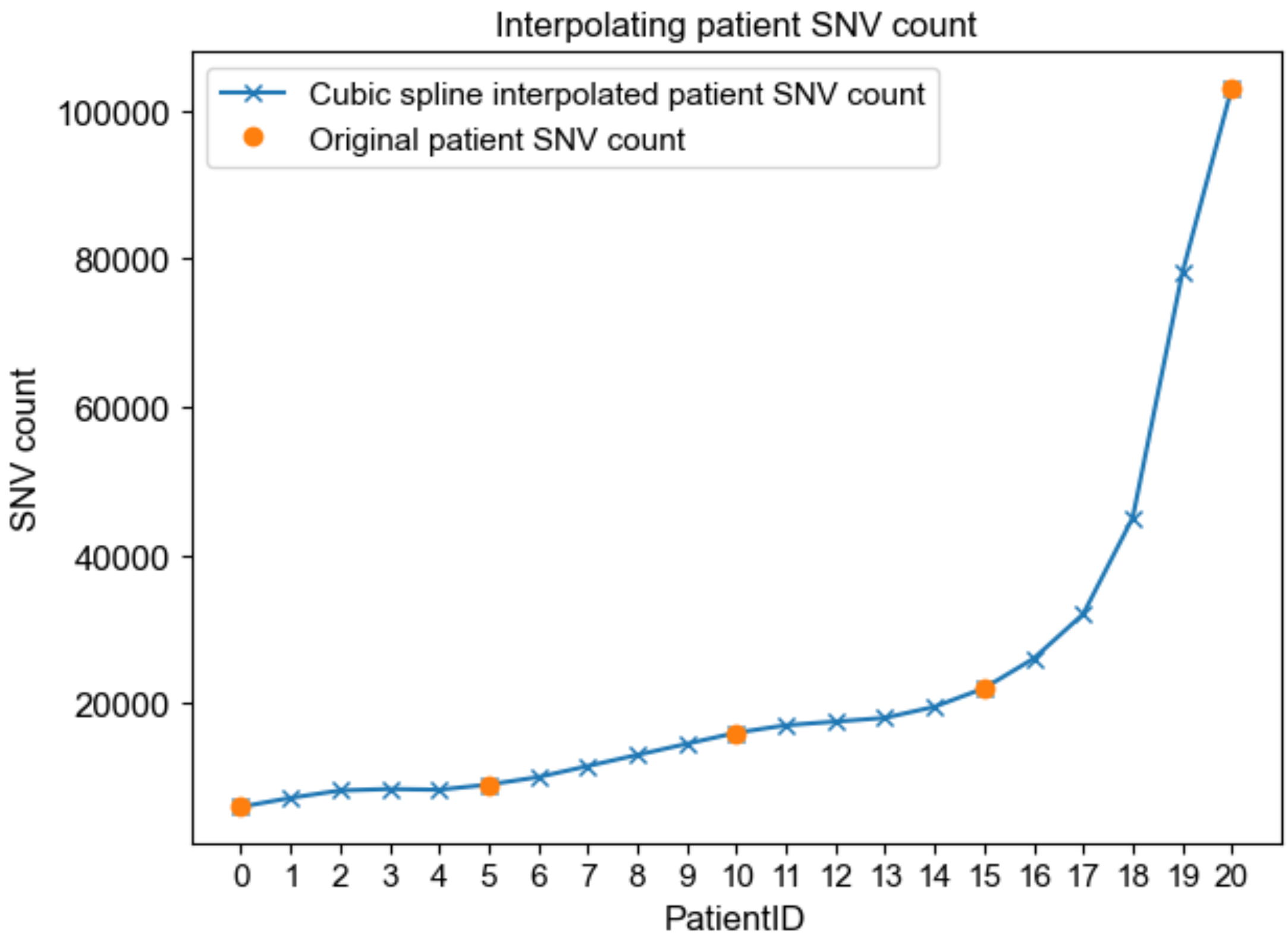

Supplementary Figure S6. Interpolation of patient SNV number in simulated patient profiles based on original PCAWG patient SNV counts in each tumor type.
Observed PCAWG patient SNV counts (orange circles) were ordered by mutation burden (SNV number) and connected by a cubic spline to generate intermediate simulated patient profiles (blue crosses), simulating the continuous progression of mutation load within each tumor type. Data shown are mock data.

Supplemental Tables

Supplemental Table 1. Interpolated patient sSNV count for all cancer types (Access via https://github.com/UMCUGenetics/cfdetect/blob/main/output/SupplTable_S1.csv, SupplTable_S1.csv).

Supplemental Table 2. t-ldTF per tumor type in all sequencing conditions (Access via https://github.com/UMCUGenetics/cfdetect/blob/main/output/SupplTable_S2.csv, SupplTable_S2.csv).

Supplemental Table 3. Similarity of interpolated SNV number distribution and original SNV number distribution with KS statistics and P-values from Kolmogorov–Smirnov test, see below.

	KS statistic	P-value
Breast-cancer	0.020	1.000
Skin-melanoma	0.020	1.000
Ovarian-cancer	0.017	1.000
Non-small-cell-lung-cancer	0.035	1.000
Colorectum-carcinoma	0.052	0.999
Stomach-cancer	0.028	1.000
Esophagus-cancer	0.037	1.000
Hepatocellular-carcinoma	0.018	1.000
Prostate-carcinoma	0.028	1.000
Pancreas-carcinoma	0.030	1.000
